# Case report: A founder UGDH variant associated with developmental epileptic encephalopathy in Saudi Arabia

**DOI:** 10.3389/fgene.2023.1294214

**Published:** 2024-01-16

**Authors:** Manal Alaamery, Salam Massadeh, Manar Aldarwish, Nour Albesher, Nora Aljawini, Othman Alahmed, Amna Kashgari, Christopher A. Walsh, Wafaa Eyaid

**Affiliations:** ^1^ Developmental Medicine Department, King Abdullah International Medical Research Center, King Saud Bin Abdulaziz University for Health Sciences, Ministry of National Guard-Health Affairs, Riyadh, Saudi Arabia; ^2^ Saudi Genome Program, National Centre for Genomic Technologies, King Abdulaziz City for Science and Technology (KACST), Riyadh, Saudi Arabia; ^3^ KACST-BWH Centre of Excellence for Biomedicine, Joint Centres of Excellence Program, King Abdulaziz City for Science and Technology (KACST), Riyadh, Saudi Arabia; ^4^ Genetics and Precision Medicine Department (GPM), King Abdullah Specialized Children’s Hospital (KASCH), King Abdulaziz Medical City, Ministry of National Guard Health Affairs (MNG-HA), Riyadh, Saudi Arabia; ^5^ King Saud bin Abdulaziz University for Health Sciences, King Abdullah International Medical Research Centre, Ministry of National Guard Health Affairs, Riyadh, Saudi Arabia; ^6^ Faculty of Sciences, King Abdulaziz University, Jeddah, Saudi Arabia; ^7^ King Saud bin Abdulaziz University for Health Sciences (KSAU-HS), King Abdullah International Medical Research Centre, Ministry of National Guard Health Affairs, Riyadh, Saudi Arabia; ^8^ Department of Radiology, King Abdullah Specialized Children’s Hospital, King Abdul Aziz Medical City, Riyadh, Saudi Arabia; ^9^ Division of Genetics and Genomics and Howard Hughes Medical Institute, Department of Pediatrics, Boston Children’s Hospital, and Departments of Neurology and Pediatrics, Harvard Medical School, Boston, MA, United States

**Keywords:** *UDP-glucose dehydrogenase*, whole-exome sequencing, gene, Saudi, encephalopathy, mutation

## Abstract

Congenital disorders of glycosylation (CDG) are a group of more than 100 rare genetic disorders characterized by impaired glycosylation of proteins and lipids. The clinical presentation of CDG varies tremendously, from single-organ to multi-organ involvement and from prenatal death to a normal adult phenotype. In this case study, we report a large consanguineous family with multiple children suffering from cerebral palsy, seizure, developmental and epileptic encephalopathy, and global developmental delay. Whole-exome sequencing (WES) analysis revealed a homozygous variant in the *UDP-glucose dehydrogenase* (*UGDH*) gene (c.950G>A; p.R317Q) which segregates with the familial phenotype with a plausible autosomal recessive mode of inheritance, indicating a potential disease-causing association. The *UGDH* gene encodes the UDP-glucose dehydrogenase, a key enzyme in the synthesis of specific extracellular matrix constituents (proteoglycans and glycolipids) involved in neural migration and connectivity during early brain development. Many pathogenic mutations of *UGDH* have been reported in recent literature works. However, the variant identified in this study has been observed only in the Saudi population (13 families) and not in any other ethnic background, suggesting that it may be an ancient founder mutation.

## Introduction

Developmental epileptic encephalopathies (DEEs) are a complex group of neurological disorders characterized by cognitive, motor, and electroencephalographic (EEG) abnormalities (Hengel et al., 2020; Jdila et al., 2021). As per the Global Burden of Disease Study 2010 (GBD), neurological disorders are responsible for approximately 3% of global disability-adjusted life years (DALYs), a term used to describe years of life lost due to premature death and disability. Epilepsy-related disorders account for 25% of these conditions (Spencer, 2013). Despite their heterogeneous phenotypes, numerous DEEs are inherited as autosomal recessive disorders, suggesting the potential increase in their risk in association with consanguinity (Strømme et al., 2010). Many studies showed that a positive family history of epilepsy contributes significantly as a risk factor for DEEs, particularly among Arabs, where consanguinity is a common practice with a prevalence ranging from 20% to 50% (Tadmouri et al., 2009). In a recent review, Idris et al. (2021) discussed the prevalence and incidence of epilepsy in Arab countries and concluded that parental consanguinity is the most frequently reported risk factor (Idris et al., 2021).

With the advancement of next-generation sequencing (NGS) technologies, researchers continue to identify many genes associated with epilepsy (Ondruskova et al., 2021; Zhang et al., 2023). However, the current next-generation sequencing (NGS) panels for epilepsy and whole-exome sequencing (WES) testing have a diagnostic yield of 25% and 45%, respectively (Krey et al., 2022). Mutations in genes associated with epilepsy cause diverse pathological defects, including ion channel dysfunction, synaptic impairment, transporter defects, and metabolic abnormalities, such as glycosylation pathway deficiencies (Freeze et al., 2015). Although some of these cases have been well-documented and discovered, some epilepsy cases remain etiologically unclear.

In this report, clinical and molecular findings in a large Saudi family with several DEE cases are presented. Whole-exome sequencing (WES) analysis identified an autosomal recessive variant in the *UDP-glucose dehydrogenase* (*UGDH*) gene associated with the DEE phenotype of the affected individuals. To evaluate the pathogenic effect of this sequence variant, an *in silico* analysis was performed using PolyPhen and SIFT. This report highlights a likely founder mutation in the Saudi population.

## Materials and methods

### Study participants

The family was referred to King Abdullah Specialist Children Hospital, Clinical Genetics and Metabolic Disorders Clinic, for the diagnosis using WES trio plus analysis for the index, affected sibling, and parents which was performed in concordance with the provisions of the Institutional Review Board (IRB) at KAIMRC, Ministry of National Guard Health Affairs (MNG-HA). Written informed consent was obtained from patients or their guardians clarifying the benefits and risks of clinical whole-exome sequencing testing.

### WES workflow

All samples were processed at Centogene’s laboratory. DNA was extracted in the Centogene facility, which is CAP- and CLIA-certified, complying with the ACMG guidelines. Full-exome capture and sequencing were performed using an Illumina platform. The sequencing libraries were enriched for target regions using the Twist Human Core Exome Plus kit. The captured libraries were sequenced to obtain at least ×20 coverage depth for >98% of the targeted bases.

### Sequence variant analysis

Sequence analysis and interpretation were performed by Centogene using an end-to-end in-house bioinformatic pipeline, including read alignment to GRCh37/hg19 ((GRCh37; (Spencer, 2013)) genome assembly, variant calling, annotation, and comprehensive variant filtering. The analysis pipeline has been previously described. Following primary filtration of low-quality reads and possible artifacts and variant annotation, detected variants were screened using several databases, including gnomAD, HGMD, ClinVar, and CentoMD. All variants with minor allele frequency (MAF) of less than 1% are considered. The search for relevant disease-causing variants is focused on coding exons and flanking ±20 intronic nucleotides of genes with a clear gene–phenotype association (OMIM information), taking into account all possible modes of inheritance. In addition, family history and clinical information were used to evaluate identified variants with respect to their pathogenicity and causality. Only variations within genes potentially related to the proband’s medical condition were reported.

## Results

**FIGURE 1:**
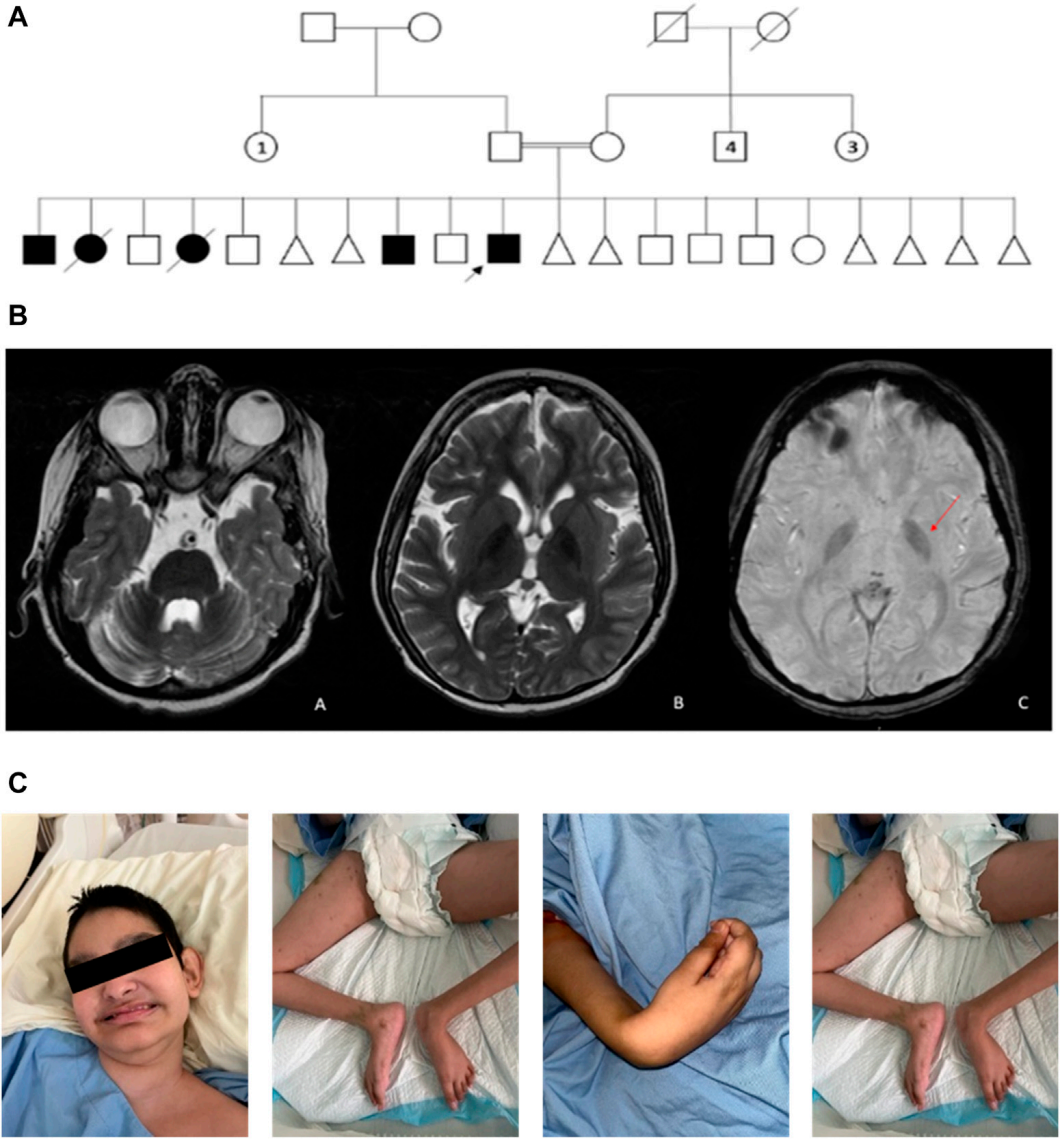
**(A)** Pedigree of a consanguineous family with multiple individuals affected with cerebral palsy and developmental and epileptic encephalopathy. The pedigree demonstrates autosomal recessive inheritance. Double lines are indicative of a consanguineous union. Circles and squares indicate females and males, respectively. Triangles indicate spontaneous abortions. Clear symbols signify healthy individuals, and filled symbols signify affected individuals. The arrow signifies the index patient. Numbered symbols indicate the number of siblings. Crossed symbols indicate deceased individuals. **(B)** MRI brain image. (a) Axial T2-WI image shows diffuse cerebellar volume loss with prominent folia and ex-vacuo dilatation of the fourth ventricle. (b) Axial 21-WI image shows mild diffuse cerebral atrophy with prominent lateral ventricles. Furthermore, there is bilateral low signal intensity in the globus pallidus. (c) Axial susceptibility-weighted image shows bilateral low signal intensity (arrow) in the globus pallidus indicative of iron overload. **(C)** Photographs of the index facial and body features show hypotonia and contractures in lower and upper limb joints.

A consanguineous Saudi family was referred to National Guard Health Affair (NGHA) hospital with multiple children affected with cerebral palsy, global developmental delay, and developmental and epileptic encephalopathy. There are three affected males, two affected females (deceased), and seven healthy children along with several aborted babies in the family (Figure 1A). The index patient is a male who was 14 years old at the time of evaluation and was born at full-term via cesarean section due to placental insufficiency. His birth weight was 2.7 kg (less than third percentile), length was 46 cm (less than third percentile), and head circumference was 33 cm (less than 3rd percentile); Apgar scores were 8 and 9 at 5 and 10 min, respectively. After delivery, he remained in the hospital for a few days with the mother and was then discharged home in good condition. The first concern by the parents was at age 1 year due to global developmental delay (GDD). At that time, he was unable to walk, crawl, or even sit without support, and he was just lying in bed.

At the age of 2 years, he developed seizures and was referred to a neurology clinic for further evaluation and management of his seizures. MRI scanning showed supra- and infratentorial parenchymal volume loss with prominent lateral ventricles (Figure 1B). The patient was referred at age 14 years to the genetic counseling service for further evaluation, where his examination showed growth parameters as follows: weight, 16.5 (less than third percentile); length, 130 cm (less than third percentile); and head circumference, 49.5 cm (less than third percentile); he did not show any dysmorphism. Neurological examination showed hypotonia and spastic quadriplegia with hyporeflexia and scoliosis (Figure 1C). Other systemic examinations were unremarkable.

His development was severely delayed as he showed poor head control, could be seated with support only, and produced sound without any words. His upper GI study showed mild gastroesophageal reflux. The milk scan showed normal gastric emptying with mild gastroesophageal reflux. The auditory brainstem response (ABR) and ophthalmology examination were normal. Skeletal survey examination demonstrated S-shaped thoracolumbar scoliotic deformity. Echocardiogram and electrocardiogram were unremarkable. Extensive biochemical investigations including ammonia level, lactic acid, creatine kinase (CK) level, total homocysteine, acylcarnitine profile, plasma amino acids, urine organic acids, and chromosomal analysis were unremarkable. Similar clinical features were remarkable in his affected siblings in the form of the observation of microcephaly, and no other significant dysmorphism at birth, developmental delay, and seizures were noted in the first 2 years. In addition, they showed scoliosis and severe global developmental delay. The living patients are bed-bound, producing random voices, and dependent on others.

**FIGURE 2:**
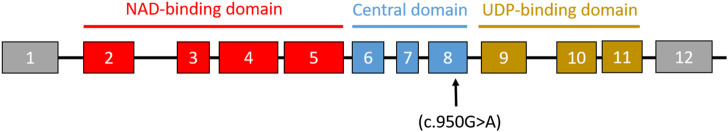
Schematic representation of the UGDH exons and domains. The arrow indicates the position of the missense mutation (c.950G>A) in the reported variant.

WES performed on the index cases revealed a rare missense variant in exon 8 of the *UGDH* gene, NM_003359.3: c.950 G>A, resulting in an arginine 317 to glutamine (R317Q) substitution at a conserved residue in the central domain (Figure 2). This variant was detected in his brother in a homozygous state. The segregation analysis revealed that this variant is inherited from healthy heterozygous parents. This UGDH variant has been documented in large-scale sequencing databases such as gnomAD, ExAC, dbSNP/1000, and ESP as an extremely rare variant with 4.89 × 10^−5^ allele frequency (gnomAD), and according to *in silico* prediction tools such as SIFT and PolyPhen, it appears to be deleterious/damaging. Moreover, *in silico* structure analysis predicted that the replacement of the negatively charged and highly conserved arginine 317 with the positively charged glutamine (R317Q) would affect the homodimerization of the UGDH core domain.

*UDP-glucose dehydrogenase* (UGDH) is a gene located on chromosome 4q15.1 that codes for an enzyme that catalyzes the conversion of UDP-glucose (UDP-Glc) to UDP-glucuronic acid (UDP-GlcA) through the simultaneous reduction of NAD^+^ to NADH. UDP-GlcA is a central player in a variety of cellular processes, including metabolism and biosynthesis. In addition to glucuronidation, UDP-GlcA is required for proper extracellular matrix formation as it is involved in the synthesis of glycans and their attachment to proteins and lipids to form glycoproteins and glycolipids. Congenital disorders of glycosylation (CDG) were first reported in 1980, and they account for more than 100 rare human genetic disorders. The majority of these metabolic diseases affect the central and/or peripheral nervous systems (Jaeken, 2013; Barone et al., 2014; Ondruskova et al., 2021). Table 1 lists all UGDH mutations associated with epileptic encephalopathy.

**TABLE 1:** List of candidate UGDH mutations associated with epileptic encephalopathy.

*UGDH* mutation	SNP	Main phenotype^a^	Clinical significance	Reference
His449Arg	rs779324355	EE	Likely pathogenic	Hengel et al. (2020)
Arg443His	rs1053767552			
Arg442Trp	rs201894374			
Ala410Ser	rs770456604			
Arg393Trp	rs113094436			
Tyr356Ter	rs1260191836			
Met306Val	rs1578265048			
Val303Ile	rs1578265068			
Gly271Arg	rs1578269200			
Ile255Thr	rs1186496501			
Glu217Asp	rs1578269761			
Pro175Ala	rs756467468			
Gln155Ter	rs1381665298			
Ile125Thr	rs1578270476			
Ile42Thr	rs1578282133			
Ala24Thr	rs1306655122			
Ala44Val	rs749975104	DEE		
Tyr367Cys	rs1578264574		Pathogenic/likely pathogenic	
Ala82Thr	rs1578274054			
Ses72Ala	rs769243823			
Arg65Ter	rs200059198			
Tyr14Cys	rs369608407			
Arg317Gln	rs775162839		Pathogenic	Hengel et al. (2020); Alhamoudi et al. (2020)

^a^ EE, epileptic encephalopathy; DEE, developmental and epileptic encephalopathy.

## Discussion

In this study, clinical and molecular characterization of a large consanguineous family of Saudi origin is presented, which manifests autosomal recessive inheritance of cerebral palsy, seizure disorder, developmental and epileptic encephalopathy, and global development delay.

Several pathogenic UGDH mutations associated with EE and DEE have been described in recent literature works (Table 1). This variant (c.950 G>A) was identified in 12 patients from 11 unrelated Saudi families with similar inheritance patterns (Alhamoudi et al., 2020; Hengel et al., 2020). The phenotypes described in homozygous carriers of this variant suggest differences (Table 2). While most cases exhibited epilepsy, two cases (one male and one female) were reported with no seizures. One epileptic case and one non-epileptic case presented dysmorphism, and seven others did not. Microcephaly was observed in two cases other than the index case in this report, and all were epileptic cases. Hypotonia and hyperreflexia are common observations. The MRI of three cases was unremarkable, while the rest of the determined cases had anomalies. Unremarkable MRI was observed in two cases with seizures and one with no observed epilepsy. Overall, the phenotype observed here shares the combined characteristics (epilepsy, microcephaly, and brain MRI anomaly) with two reported cases and differs in degree from the rest of the reported cases.

**TABLE 2:** Summary of the (c.950G>A) mutation-associated phenotype.

Patient information	Reported phenotype	Age of observation	Reference
Family 16 II: 1 female	Daily generalized tonic and myoclonic seizures	12 months	Hengel et al. (2020)
	Generalized hypotonia, with contractures and hyperreflexia at upper and lower extremities		
Family 17 II: 2 female	No epilepsy	5 years	
	Generalized hypotonia		
Family 18 II: 1 female	Daily generalized tonic and clonic and later myoclonic seizures with eye fluttering	20 months	
	Generalized hypotonia and hyperreflexia at upper and lower extremities with clonus and positive Babinski		
Family 19 II: 1 female	Recurrent generalized tonic and clonic convulsions	30 months	
	Axial hypotonia and peripheral hypertonia and hyperreflexia in upper and lower limbs		
	MRI: mild thinning of the corpus callosum and a decrease in white matter volume bilaterally	2 years	
Family 19 II: 2 female	Daily myoclonic seizures with eye fluttering	18 months	
	Generalized hypotonia and hyperreflexia at upper and lower extremities with clonus and positive Babinski		
Index case male	No seizures	13 months	Alhamoudi et al. (2020)
	Delayed developmental skills, global joint hyperlaxity, and axial hypotonia		
	Subtle dysmorphic features, including bifrontal narrowing, bulbous nose, smooth philtrum, and high-arched palate		
	Sit without support and follow simple commands	5 years	
	Axial hypotonia with some weakness in the muscles		
	MRI: unremarkable		
Index case male	Global developmental delay	1 year	This report
	Seizures	2 years	
	MRI: supra- and infratentorial parenchymal volume loss with prominent lateral ventricles		
	Hypotonia and spastic quadriplegia with hyporeflexia and scoliosis	14 years	
	Poor head control, could be seated with support only, and produced sound without any words		

Homozygous carriers of the UGDH variant reported in this study have been observed only in individuals of Saudi descent. Considering that all reported patients belong to the same ethnic group and exhibit the same mutation and inheritance pattern, our study supports that the identified pathogenic variant (c.950 G>A) causes UGDH-associated developmental and epileptic encephalopathy and that this variant is a founder mutation in the Saudi population, highlighting the profound effect of consanguinity on offspring morbidity and mortality.

Due to the heterogeneity of developmental and epileptic encephalopathies (DEEs), the identification of the genetic basis and electroclinical syndromes is crucial for the development of a care approach (Lee et al., 2014). A wide range of interventions, including medications, surgery, and dietary restriction, is used to increase quality of life, improve seizure control, and relieve or circumvent related comorbidities (Vasquez et al., 2022). Precision therapies for DEE are emerging that target a specific pathogenesis mechanism or restore a defective gene function (Vasquez et al., 2022). Early detection and treatment may result in better neurocognitive performance, illustrating the importance of genetic testing and research (Lee et al., 2014). A small number of UGDH-associated DEE patients benefit from existing treatments such as sodium valproate and ketogenic diet (Hengel et al., 2020). Therefore, more effort should be invested in employing emerging gene-targeting technologies such as antisense oligonucleotide modulation and gene therapy for UGDH DEE patients.

## Data Availability

The data presented in the study are deposited in the LOVD 3.0 repository, accession number 0044213. More information can be found at https://databases.lovd.nl/shared/individuals/00442132, https://databases.lovd.nl/shared/variants/0000946886.
